# Extender Supplementation with Glutathione (GSH) and Taurine Improves *In Vitro* Sperm Quality and Antioxidant Status of New Zealand Rabbits during Chilled Storage for up to 72 hours

**DOI:** 10.1155/2023/8339591

**Published:** 2023-09-12

**Authors:** Mohamed F. F. Bayomy, Sobhy E. Hassab El-Nabi, Tahany A. El Kassas, Zeinab I. Attia, Ayman M. Saeed, Heba S. A. Taha, Mahmoud Alagawany, Livio Galosi, Lucia Biagini, Seham El-Kassas

**Affiliations:** ^1^Zoology Department, Faculty of Science, Menoufia University, Shibin Al Kawm, Egypt; ^2^General Biology Department, Center of the Basic Sciences, Misr University for Science and Technology (MUST), 6th of October City, Egypt; ^3^Zoology Department, Faculty of Science, Tanta University, Tanta, Egypt; ^4^Biotechnology Department, Animal Production Research Institute, Giza, Egypt; ^5^Department of Genetics, Faculty of Agriculture, Zagazig University, Zagazig, Egypt; ^6^Poultry Department, Agriculture Faculty, Zagazig University, Zagazig 44519, Egypt; ^7^School of Bioscience and Veterinary Medicine, University of Camerino, Matelica 62024, Italy; ^8^Animal, Poultry, and Fish Breeding and Production, Department of Animal Wealth Development, Faculty of Veterinary Medicine, Kafrelsheikh University, Kafr el-Sheikh, Egypt

## Abstract

This study assessed the influence of supplementing the rabbit semen extender with various concentrations of glutathione (GSH) and taurine at 24, 48, and 72 h postchilling at 5°C. Semen samples were collected from 20 New Zealand bucks, and ejaculates with standard color, motility (>85%), about 0.5 mL volume, and ∼400 × 10^6^/mL concentration were used and diluted with extenders supplemented with 0.5, 1, and 2 mM of GSH and 1, 5, and 10 mM of taurine and chilled at 5°C. Nonsupplemented samples were used as a control. Sperm's progressive motility, acrosome reaction, and extracellular oxidative stress biomarkers such as MDA contents and GPx, SOD, and CAT concentrations and intracellular transcriptomic levels of *SOD* and *CAT* genes were assessed. GSH and taurine supplementation improved the sperm's kinetics by reducing cooling-associated stress, which was ascertained by lowering MDA concentration and increasing SOD, CAT, and GPx concentrations (*P* < 0.05). Increasing the levels of antioxidant enzymes in the extender was due to the increasing mRNA copies of the *SOD* and *CAT* genes (*P* < 0.05). Furthermore, GSH and taurine maintained the fructose levels in the extender and lowered the GPT levels, which implies sperm membrane stability is maintained through GSH and taurine supplementation. GSH and taurine supplementation to the extender had protective influences on the *in vitro* rabbit semen quality during chilled storage for up to 72 h, which were remarkable with increasing supplementation dose and cooling time at 5°C.

## 1. Introduction

With the increase in human population and the decrease in resources, especially with the global climatic changes, ensuring food and nutritional security is a fundamental requirement [1–5]. Thus, increasing animal productivity is necessary, and this goal can be achieved by increasing the number of animals, breeds, and strains produced. Artificial insemination (AI) has been proven to be an efficient tool to improve the animal's genetic potential, increase its yielding capacity, and selectively increase genetic gain through increasing male selection intensity [6].

Rabbit is an indispensable animal species to meet the nutritional requirements of human beings and for biomedical research. Rabbits are generally used as valuable meat sources due to their high feed conversion rate and fur production, and they are also used as pets [7]. Besides, in biomedical research, this species is for antibody production and as a model for human diseases such as hypercholesterolemia and atherosclerosis [8]. Moreover, gene-modified rabbits were usefully developed and used for human disease research using genetic engineering applications such as gene editing tools, gene knockout, and conventional pronuclear microinjection techniques [9, 10]. Thus, it is vital to maintain and increase the production of rabbit strains for these many purposes. Preserving rabbit strains can be achieved by repeated breeding, but numerous animals must avoid inbreeding depression [11, 12]. Another way is the cryopreservation of ova and embryos in liquid nitrogen, which requires scarifying animals, professional surgical skills, and a high cost [10]. Therefore, cryopreservation of semen by repeated collection and preservation in liquid nitrogen is more effective [10], but most rabbit production relies on using fresh or shortly stored semen for routine AI [13]. However, the oxidative stress caused by the excessive production of reactive oxygen species (ROS) [14–20], and the consequent cellular, cytoplasm, and genome damage alter sperm quality [21]. Thus, the effectiveness of AI using cooling semen depends on the ability of the extenders to provide a suitable environment for spermatozoa metabolism.

AI programs in rabbits are currently performed with fresh diluted semen collected within 6–18 hours, and bucks are typically kept on the same farm [22]. Improving semen storage capacity would allow for a longer interval between semen collection and female insemination, thereby improving AI performance and allowing the existence of farms without males [23, 24]. However, storing rabbit sperm for more than 24 to 48 hours decreases fertility [25–27]. Therefore, one of the primary goals is to extend the interval of liquid semen storage beyond 48 hours or to freeze the semen. Although satisfactory in-vitro results on sperm quality and fertilizing ability have been obtained with frozen semen in the last decade [28–30], the limited survival of sperm after freezing is a major drawback for the widespread use of frozen semen in AI programs. Semen cryopreservation induces damage to all sperm compartments related to the intra- and extra-ice formation and causes a ROS excess generated by dysfunctional mitochondria, responsible for sperm membrane injuries [10, 31–34]. These damages are responsible for the loss of sperm motility, viability, acrosomal, and DNA integrity which decreases the fertilizing capacity of frozen-thawed sperm [35]. Moreover, the freezing technique is more expensive and requires more equipment and technical expertise than the chilling technique. AI with cryopreserved rabbit sperm finds applications in experimental or genetic resource banking. Under this view, chilling semen between 24 and 48 hours is still essential and is applicable, as this technique is simple and inexpensive, requiring no special equipment. Accordingly, more attempts are needed to improve semen extenders and storage conditions to prolong the time stored semen can maintain its functional status.

Many approaches have been utilized to preserve and improve semen quality and sperm viability, including the supplementation of antioxidant substances, such as glutathione (GSH), taurine, lycopene, cysteine, and glutamine directly to the animals or in semen extenders to increase the antioxidant abilities and lower the impacts of oxidative stress [36, 37]. GSH and taurine are the most common antioxidants, which have markedly improved the antioxidant characteristics of semen extenders. GSH maintains semen quality by keeping better integrity of sperm's nuclear and plasma membranes. Besides, it improves the fertilizing ability of spermatozoa following the freezing-thawing process [38]. The improving effects of GSH are due to its property as an electron donor to reduce the H_2_O_2_ production plus its oxidation to glutathione disulfide (GSSG). Moreover, the thiol group content of the amino acid cysteine in GSH penetrates the spermatozoa's plasma membrane enhancing the intracellular biosynthesis of GSH, which protects sperm's DNA, proteins, and its membrane-lipids content via scavenging of ROS [39]. The sulfonic amino acid taurine is a nonenzymatic scavenger that also protects the spermatozoa by inhibiting lipid peroxidation and protecting sperm cells against the accumulation of ROS [37]. These antioxidant properties of GSH and taurine may be allied to modification in the levels of antioxidant enzymes such as sodium oxide dismutase (SOD) and catalase (CAT) which in turn may be linked with the changes in the transcription levels of their genes. Thus, this investigation examined the mRNA levels of SOD and CAT genes. Additionally, taurine enhances the motility of spermatozoa by protecting the integrity of the plasma, nuclear, and mitochondrial membranes and maintaining the structure of the cytoskeleton of the flagella of spermatozoa [40].

In the previous study of Ahmad et al, the exogenous antioxidants supplementation of GSH at 0, 1, 2, 4, and 8 mM to rabbit semen extender stored at 5°C for 24 h improved the semen attributes, especially the lower doses [41]. However, to the best of our knowledge, no reports on the GSH effect on semen attributes using lower doses and longer storage times. Also, It has been shown that supplementing taurine at 0, 1.5, 7, 12.5, and 50 mM in a semen extender and stored at 37°C for 4 h did not alter the viability, morphology, and acrosome integrity of rabbit spermatozoa [42]. Therefore, it was recommended to use taurine in rabbit extender at lower temperatures for possible protective effects. Therefore, the current study aimed to test the effect of supplementing different concentrations of GSH and taurine on the antioxidant properties of semen extender and semen quality after different chilling times.

## 2. Materials and Methods

### 2.1. Animal Management and Semen Collection

The current study was conducted following the ethical and regulation procedures of the Department of Animal Wealth Development, Faculty of Veterinary Medicine, Kafrelsheikh University, Egypt (approval number: KFS-2021/05).

This study used 20 rabbit bucks (*n* = 20) of the New Zealand white rabbit breed (about 2-3 kg body weight and 1–1.5-year-old). Bucks were individually labeled and caged with free access to feed and water. Animals were housed in well-ventilated rooms with a natural daylight supply. Bucks were trained for two weeks for semen collection using an artificial vagina and a mature female as a mount stimulator. Then, ten ejaculates from each buck were collected (one ejaculate/week), during the ten-week trial, for a total of 200 ejaculates. In each collection, fresh semen samples were microscopically examined and evaluated after removing the gel clot. The spermatozoa activity was microscopically evaluated at 100x (to evaluate mass motility) and 400x (for individual motility) using a phase-contrast microscope (Micros Austria, Micros St. Veit Hunnenbrunn–Gewerbezone, Veit/Glan, Austria) [43]. A Neubauer hemocytometer was used to measure sperm concentration. Only ejaculates with a standard color, more than 85% motility, about 0.5 mL volume, and ∼400 × 10^6^/mL concentration were used for the study. During each time of collection, ejaculates were pooled (*n* = 20 with an average volume of ejaculates ∼ 8.75 ± 1.165 mL) to avoid individual variations and get sufficient semen volume for different treatments [41].

### 2.2. Extender Preparation and Semen Processing and Evaluation

The pooled semen samples were diluted in a 1 : 2 basic extender [41] containing 20 mM Tris, 57 mM citric acid anhydrous, 5 mM fructose, 22 mM glucose, and 4.6 mM streptomycin along with 10 mL egg yolk and completed to 100 mL with distilled water with pH 6.8–7, and osmotic pressure was kept at pH 6.9, 300 mOsm. The diluted pooled semen samples were divided equally into 7 fractions (1 fraction/treatment). The diluted semen samples were then supplemented with 0 (control with diluent only without additives), 0.5, 1, and 2 mM glutathione (GSH; cat. no. Y0000517, Merck KGaA, Darmstadt, Germany) and 1, 5, and 10 mM taurine (cat. no. T0625, Merck KGaA, Darmstadt, Germany). The pH was adjusted to 6.8–7 with a dilution factor of 1 : 2 (semen: prepared extenders), and the diluted semen was kept at 5°C. Then, changes in semen quality were evaluated after 24, 48, and 72 h of chilling. Sperm motility, viability (% of live spermatozoa), acrosome reaction, fructose and glutamic pyruvic transaminase (GPT) concentrations, and antioxidant enzymes activity (catalase (CAT), superoxide dismutase (SOD), GSH, and malondialdehyde (MDA)) as oxidative biomarkers were assessed. Relative transcriptomic levels of SOD and CAT genes were also evaluated. All the evaluations were repeated ten times throughout the trial (ten weeks).

### 2.3. Assessment of Sperm's Viability and Progressive Motility

Sperm viability was evaluated for control and treated samples according to the literature [41]. Sperms were stained with propidium iodide (PI, 200 *μ*g/mL semen), and viability was morphologically assessed based on sperm's membrane integrity differences: sperms with damaged membrane were penetrated by PI stain showing partial or complete pinkish fluorescence, which indicates dead sperm. At the same time, the live sperm exhibited no fluorescence over the sperm head. Slides were examined at 1,000x magnification using a phase‐contrast microscope (Leica Microsystems Inc., Parkway North, Deerfield, IL 60015 United States), and 200 sperms/slide were counted and evaluated.

Sperms' progressive motility was evaluated by visually measuring the percentage of spermatozoa showing any movement of the flagellum using a phase-contrast microscope (Micros Austria, Micros St.Veit Hunnenbrunn–Gewerbezone, Veit/Glan, Austria), that was equipped with a warm stage. The motility was measured after 24, 48, and 72 h of chilling for control and different treatments. Briefly, 5 *μ*L of every semen sample was incubated at 37°C for 20 min, placed on a prewarmed glass slide, and covered by a glass cover slider. Slides were examined at 400x. Sperm motility was assessed in five microscopic fields for each semen sample (in the five examined fields within 1 min). Sperm motility was evaluated as the percentage of spermatozoa that moved their flagellum. The average of the five fields was used to calculate the final sperm motility value [41, 44].

### 2.4. Analysis of Acrosome Reaction

Acrosome reaction was also evaluated after 24, 48, and 72 h of chilling in differently treated semen samples using Giemsa stain according to Watson's methods with some modifications [45]. Briefly, 30 *μ*L from each semen sample was smeared on glass slides, air-dried, fixed with 10% neutral formal saline for 15 min, washed in running water for 20 min, and allowed to dry. After drying, slides were immersed in Giemsa stain solution overnight. After incubation, the slides were rinsed with distilled water and air-dried. The acrosome status of 100 spermatozoa for each sample was examined at 1000x in each stained smear, using a phase-contrast light microscope (Micros Austria, Micros St. Veit Hunnenbrunn–Gewerbezone, Veit/Glan, Austria). Purple heads characterized spermatozoa with intact acrosomes, whereas the damaged-acrosome spermatozoa had a pale lavender head. The percentage of acrosome-intact spermatozoa was calculated.

### 2.5. Assessing the Oxidative Stress Biomarkers in Seminal Plasma

Semen samples at 24, 48, and 72 postchilling were centrifuged at 1,000 × g for 20 min, and the supernatant, composed of semen extender and seminal fluid, was used to assess oxidative stress biomarkers, using MDA contents and assessing the concentration of glutathione peroxidase (GPx), SOD, and CAT. Pellets of spermatozoa were washed by suspending them in cold fresh phosphate buffer saline, centrifuged at 3,000 × g for 10 min, and then frozen in liquid nitrogen for RNA extraction. Lipid peroxidation was evaluated by quantifying the MDA content (nmol/mL) using a commercial kit (MD 2529, Bio-Diagnostic, Egypt, with a sensitivity of 0.1 nmol/ml and detection range up to 50 nmol/ml) according to manufacturer instructions. MDA was measured with 2-thiobarbituric acid, monitoring the absorbance change at 532 nm with the spectrophotometer (Shanghai Spectrophotometer Co. Ltd., China). Antioxidant concentrations of CAT, SOD, and GPx were determined with commercial kits: CAT (CA 2517, Bio-Diagnostic, Egypt, with a sensitivity of 0.22 U/ml and detection range up to 200 U/ml), SOD (SOD 2521, Bio-Diagnostic, Egypt, with a sensitivity of 2.0 mU/ml and detection range up to 500 U/ml), and GPx (1.11.1.9, Bio-Assay Systems, United States, with a sensitivity of 1 mU/ml and detection range up to 500 mU/ml), according to manufacturer's instruction.

### 2.6. qRT-PCR of Relative Transcriptomic Levels of SOD and CAT Genes

Total RNA was extracted using TRI Reagent (Bioline, UK) following the manufacturer's protocol. Diluted semen samples were centrifuged at 3,000 × g for 20 min to eliminate semen extender and seminal fluid. After that, a pellet of spermatozoa was washed many times using ice-cold PBS. Lysis buffer was added to the sperm pellet and kept at room temperature for 10 min. Then, 1 ml TRI reagent was added, and extraction was completed. Afterward, complementary DNA (cDNA) was synthesized by reverse transcription reactions by iNtRON Biotechnology cDNA kits (Power cDNA Synthesis Kit, cat. no. 25011, iNtRON Bio, Seongnam, Gyeonggi 462-120, US) according to the manufacturer protocol. Specific primers for antioxidant-related genes such as CAT, SOD, and glyceraldehyde-3-phosphate dehydrogenase (GAPDH) genes, as shown in Table 1, were used. The amplification reaction was performed using the SensiFast SYBR Lo-Rox kit (Bioline, UK) in the Stratagene MX3000P real-time PCR system. 20 *μ*l as a total reaction volume contained 10 *μ*l SYBER green master mix, 2 *μ*l cDNA, primers 0.8 *μ*l (50 nm), and 6.4 *μ*l RNase/DNase-free H_2_O [47]. The cycling conditions were preheating at 62°C for 1 min, 95°C for 5 min, and 40 cycles at 95°C for 2 sec, and the annealing temperatures are listed in Table 1. At the end of the PCR reaction, a melting curve with 0.5°C increments in the temperature range of 65–95°C was performed to ensure the specificity of the primers. Also, amplification efficiency for each primer was estimated using the formula *E* = 100 × (10^−1/slope^-1). Samples for each treatment were repeated twice. The relative mRNA expression of the genes was calculated using the comparative threshold cycle method 2^−ΔΔCT^ [48], and the results were reported as fold change differences relative to the control group.

### 2.7. Statistical Analysis

Before data analysis, normality testing was performed by testing the Gaussian normal distribution with the Shapiro–Wilk normality test. Results of the current study were statistically analyzed using two-way ANOVA to test the effect of GSH and taurine supplementation, chilling time, and their interactions on semen quality, followed by Tukey's multiple comparison test using GraphPad Prism (GraphPrism© Software, La Jolla, CA, USA) [49–57]. Results were expressed as means ± SEM and considered significantly different at *P* < 0.05.

## 3. Results

### 3.1. Impact of GSH and Taurine Supplementation on Sperm Kinetics at Different Postchilling Times

Table 2 displays the postchilling kinetics of rabbit sperm supplemented with GSH and taurine at different concentrations (0.5–2 mM GSH and 1–10 mM taurine). The addition of GSH and taurine induced improving effects of sperm kinetic, especially with increasing chilling time. At 24 h postchilling, adding GSH and taurine at different rates did not alter sperm kinetics. However, at 48 and 72 h postchilling, there was a noticeable reduction in percentages of motile sperm, % of live sperms, and % of the acrosome reaction in nontreated semen samples. An interesting improvement of these parameters was reported for treated semen samples with GSH and taurine (*P* < 0.05).

### 3.2. GSH and Taurine Supplementation Improved the Antioxidant Properties of Semen Extender by Altering the MDA Content and Levels of Antioxidant Enzymes

**Figure 1:**
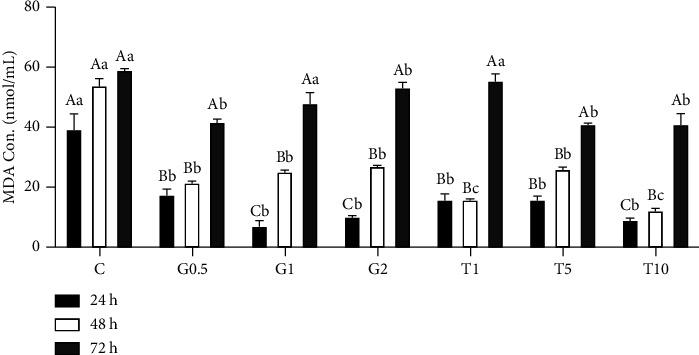
MDA contents in semen samples following different concentrations of GSH and taurine and chilling for up to 72 h. C represents the control samples (no treatment) stored at 5°C for 24, 45, and 72 h, and G1, G2, and G0.5 represent 1, 2, and 0.5 mM of GSH treatments stored at 5°C for 24, 45, and 72 h, respectively. T1, T5, and T10 denote 1, 5, and 10 mM of taurine supplementation after different postchilling times (24, 48, and 72 h) at 5°C. Different uppercase and lowercase letters indicate statistical significances at *P* < 0.05 between different chilling times and extender supplements (GSH and taurine), respectively.

Figure 1 illustrates the MDA content in semen samples following GSH and taurine supplementation after different postchilling times. GSH and taurine supplementation at different concentrations was significantly correlated with lowering MDA concentration in semen samples preserved at 5°C for 24, 48, and 72 h (*P* < 0.05). At 24 h postchilling, 1, 5, and 10 mM taurine and 0.5, 1, and 2 mM GSH-supplemented semen samples had the lowest MDA content (*P* < 0.05). After 48 h of chilling at 5°C, all samples had less MDA content than those after 24 h of chilling (*P* < 0.05). However, 1 and 10 mM taurine supplementation had the lowest level of MDA compared to the other treatments and control (*P* < 0.05). Increasing the chilling time to 72 h was significantly associated with reducing MDA content in all treatments compared to the control (except 5 and 10 mM taurine as well as 0.5 mM GSH). GSH and taurine supplementation lowers oxidative stress in chilled semen samples, up to 72 h, by reducing lipid peroxidation, confirmed by decreasing MDA contents.

**Figure 2:**
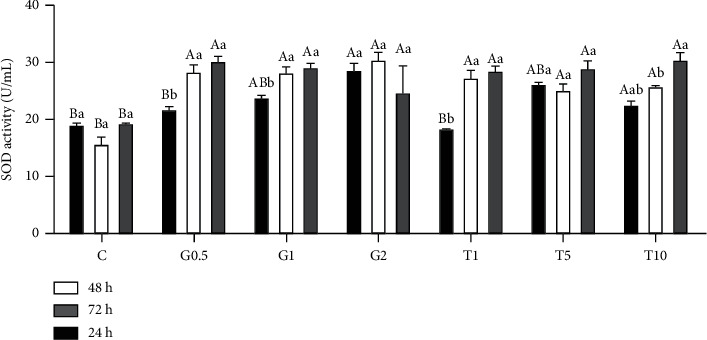
SOD activity in response to different concentrations of GSH and taurine and chilling for up to 72 h. C represents the control samples (no treatment) stored at 5°C for 24, 45, and 72 h, and G1, G2, and G0.5 represent 1, 2, and 0.5 mM of GSH treatments stored at 5°C for 24, 45, and 72 h, respectively. T1, T5, and T10 denote 1, 5, and 10 mM of taurine supplementation after different postchilling times (24, 48, and 72 h) at 5°C. Different uppercase and lowercase letters indicate statistical significances at *P* < 0.05 between different chilling times and extender supplements (GSH and taurine), respectively.

Figure 2 displays the alterations in SOD activities during different chilling times and GSH and taurine supplementation. Both chilling time and GSH and taurine supplementation markedly modified SOD activities in semen with distinct interaction (*P* < 0.05). After 24 h chilling, higher concentrations of GSH and taurine (1 and 2 mM GSH, and 5 and 10 mM taurine) significantly increased SOD activities in semen samples, compared to the lower concentration (0.5-mM GSH and 1 mM taurine) and nonsupplemented semen (*P* < 0.05). Increasing the chilling time to 48 and 72 h was associated with increasing SOD in all GSH and taurine-supplemented samples, compared to nonsupplemented semen samples (*P* < 0.05). GSH and taurine supplementation in semen extender were associated with increasing SOD and increased chilling time.

**Figure 3:**
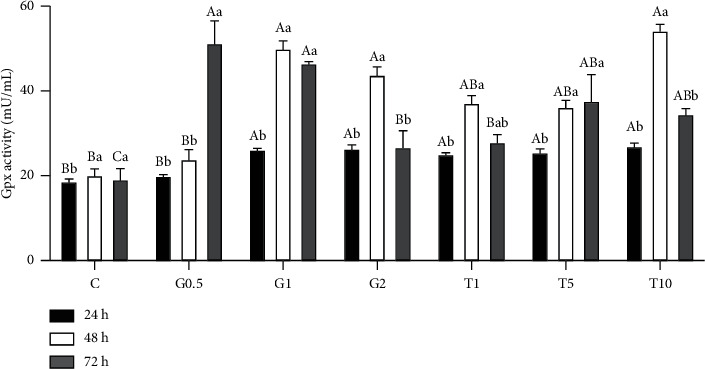
GPx activity in response to different concentrations of GSH and taurine and chilling for up to 72 h. C represents the control samples (no treatment) stored at 5°C for 24, 45, and 72 h, and G1, G2, and G0.5 represent 1, 2, and 0.5 mM of GSH treatments stored at 5°C for 24, 45, and 72 h, respectively. T1, T5, and T10 denote 1, 5, and 10 mM of taurine supplementation after different postchilling times (24, 48, and 72 h) at 5°C. Different uppercase and lowercase letters indicate statistical significances at *P* < 0.05 between different chilling times and extender supplements (GSH and taurine), respectively.

GPx activity in semen samples was also measured at different chilling times, and GSH and taurine supplementation (Figure 3). Nonsupplemented semen samples exhibited nearly fixed GPx levels over increasing chilling time. However, supplementing the semen extender with either GSH or taurine at a different concentration significantly modified GPx levels which were prominent with increasing chilling time (*P* < 0.05). In this context, all supplementing taurine and GSH doses except 0.5 mM GSH prominently increased GPx levels at 24 h postchilling. The same effect was also reported at 48 h and 72 h postchilling for all taurine and GSH doses except 0.5 mM GSH, which only was able to induce higher GPx after 72 h of chilling (*P* < 0.05).

**Figure 4:**
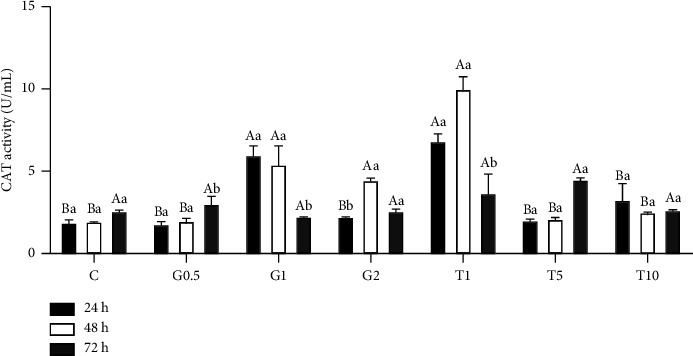
CAT activity in response to different concentrations of GSH and taurine and chilling for up to 72 h. C represents the control samples (no treatment) stored at 5°C for 24, 45, and 72 h, and G1, G2, and G0.5 represent 1, 2, and 0.5 mM of GSH treatments stored at 5°C for 24, 45, and 72 h, respectively. T1, T5, and T10 denote 1, 5, and 10 mM of taurine supplementation after different postchilling times (24, 48, and 72 h) at 5°C. Different uppercase and lowercase letters indicate statistical significances at *P* < 0.05 between different chilling times and extender supplements (GSH and taurine), respectively.

CAT activities in supplemented semen samples and at different chilling times were evaluated (Figure 4). After 24 h of chilling, 1 mM GSH and the same taurine dose induced higher levels of CAT than the other supplements and control (*P* < 0.05). Increasing chilling time to 48 h was associated with higher activities of CAT in the samples supplemented with 1 mM and 2 mM GSH along with the 1 mM taurine compared to the other treated samples (*P* < 0.05). Nevertheless, at 72 h postchilling, all treatments had nearly the same CAT activities. Additionally, the effect of GSH and taurine supplementation was significantly modified by chilling time. The highest activities of CAT were measured at 24 h and 48 h postchilling in the case of 1 mM GSH and taurine. At the same time, no differences were noticed for the other supplemented groups.

### 3.3. GSH and Taurine Supplementation to Semen-Extender Maintained Fructose and Glutamic Pyruvic Transaminase (GPT) Concentrations at Different Postchilling Times

**Figure 5:**
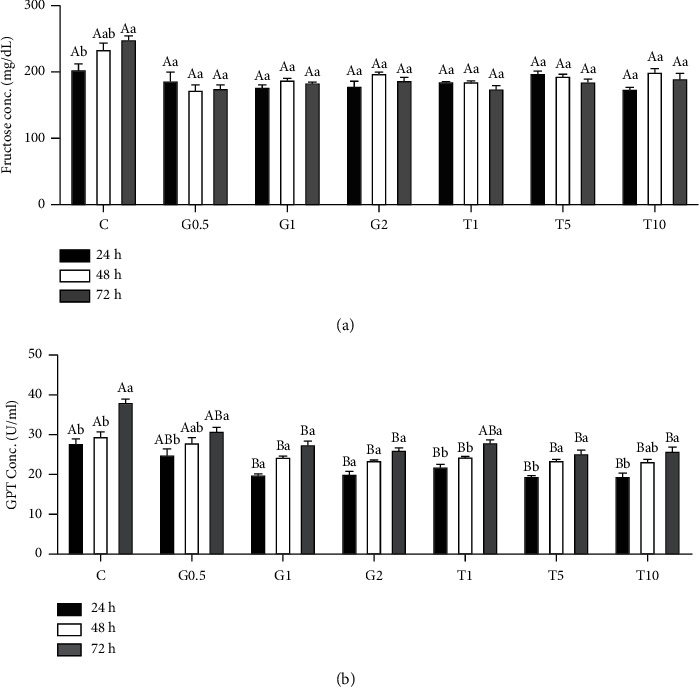
Fructose (a) and GPT (b) levels in semen samples in response to different concentrations of GSH and taurine and chill for up to 72 h. C represents the control samples (no treatment) stored at 5°C for 24, 45, and 72 h, and G1, G2, and G0.5 represent 1, 2, and 0.5 mM of GSH treatments stored at 5°C for 24, 45, and 72 h, respectively. T1, T5, and T10 denote 1, 5, and 10 mM of taurine supplementation after different postchilling times (24, 48, and 72 h) at 5°C. Different uppercase and lowercase letters indicate statistical significances at *P* < 0.05 between different chilling times and extender supplements (GSH and taurine), respectively.

Figure 5 displays changes in fructose and GPT concentrations at different chilling times, with and without GSH and taurine supplementation in the semen extender. Fructose concentration increased with increasing chilling time in the nonsupplemented semen extender (*P* < 0.05). Conversely, semen extenders supplemented with different doses of GSH and taurine showed slight changes in fructose levels at different chilling times. At 48 and 72 h postchilling, the fructose levels in nonsupplemented semen extender (control) were significantly higher than those in supplemented ones, especially if compared with those treated with 5 and 10 mM taurine (*P* < 0.05).

GPT levels were also influenced by supplementations of semen extender with GSH and taurine and chilling time (*P* < 0.05). In comparison with the nonsupplemented group (control), all supplemented semen extenders, either by GSH or taurine (at different doses), had significantly fewer GPT levels (*P* < 005). However, no significant differences in GPT levels were found among supplemented semen extenders. Besides, the GPT level in nonsupplemented extenders significantly increased with increasing chilling times (*P* < 0.05). However, it remained unchanged in the case of supplemented extenders with either GSH or taurine.

### 3.4. Relative mRNA Levels of SOD and CAT Genes at Different Postchilling Times and following GSH and Taurine Supplementation

**Figure 6:**
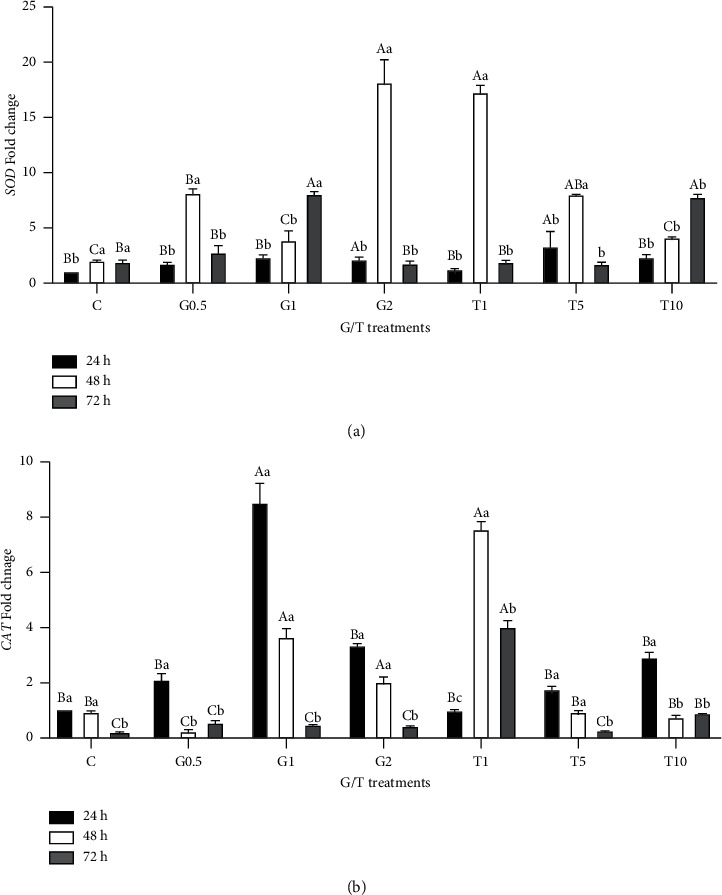
The relative expression level of SOD and CAT in sperms in response to different concentrations of GSH and taurine and chilling for up to 72 h. C represents the control samples (no treatment) stored at 5°C for 24, 45, and 72 h, and G1, G2, and G0.5 represent 1, 2, and 0.5 mM of GSH treatments stored at 5°C for 24, 45, and 72 h, respectively. T1, T5, and T10 denote 1, 5, and 10 mM of taurine supplementation after different postchilling times (24, 48, and 72 h) at 5°C. Different uppercase and lowercase letters indicate statistical significances at *P* < 0.05 between different chilling times and extender supplements (GSH and taurine), respectively.

Figure 6 illustrates the relative mRNA copies of both SOD and CAT genes in spermatozoa after different chilling times and GSH or taurine supplementation. For the SOD gene, compared to nonsupplemented semen and after 24 h cooling, increasing chilling time to 48 and 72 h was associated with significant increases in mRNA copies. This effect was also reported, especially at 48 h postchilling, for semen samples supplemented with different doses of either GSH or taurine (*P* < 0.05). The highest expression levels of SOD were determined in the case of 2 mM GSH and 1 mM taurine, followed by 0.5 mM and 5 mM taurine. Increasing the cooling time to 72 h was associated with higher expression levels of SOD, especially in the case of 1 mM GSH and 10 mM taurine, compared with nonsupplemented or GSH- and taurine-supplemented samples (*P* < 0.05).

The CAT gene's relative expression level was modulated with GSH/taurine supplementation and cooling times. Increasing chilling time to 72 h was linked with a significant reduction of CAT mRNA copies in samples either nonsupplemented or supplemented with GSH or taurine (*P* < 0.05), except for the integration with 1 mM taurine. The highest expression levels were measured at 24 and 48 h postchilling for both treated (except 1-mM taurine) or untreated samples. The expression level fluctuated with different GSH supplementation doses; the highest level was found in the case of 1 mM GSH compared to 2 mM and 0.5 mM GSH, while a different response was reported for taurine supplementation. Increasing the taurine dose was associated with lowering CAT mRNA levels at 48 and 72 h postchilling. At 24 h postchilling, minor increases were found with increasing taurine dose.

## 4. Discussion

Cooling and freezing/thawing procedures of semen markedly alter the quality of preserved sperm. In this regard, long-term cooling deteriorates sperm quality and, consequently, its fertilizing ability because of the excessive release of ROS such as superoxide anion radicals (O_2_^−^), hydrogen peroxide (H_2_O_2_), and lipid hydroperoxides [22, 58, 59]. Moreover, semen dilution during chilled storage reduces the normal physiological antioxidants included in the semen. The maintenance of the fertilizing capacity of rabbit semen for longer than 48 to 72 hours remains an important target for rabbit production because the farming system is based exclusively on AI programs, which are currently performed with fresh diluted semen within 6–12 hours from the collection. To improve semen extenders' efficacy and functionality, in the current study, we targeted increasing rabbit semen quality by GSH and taurine supplementation and tested their effects over different chilling times (24, 48, and 72 h).

Supplementing the extender with antioxidant substances such as GSH and taurine improved the semen quality, as confirmed by the significant increase in the percentages of live spermatozoa, motile sperms, and acrosome reactions. However, at 24 h postchilling, GSH and taurine did not alter these parameters compared to the control. This effect is probably related to the natural antioxidant potency of sperm to scavenge ROS production [41]. The positive effects of both GSH and taurine are increased with the supplementation dose and cooling time (at 48 and 72 h postchilling). This response might be linked to the antioxidant properties of both GSH and taurine that protect the sperm membrane from cooling-associated oxidative stress and the overproduction of ROS [58]. Spermatozoa have antioxidant defenses against the production of ROS, but dilution in semen extender and cooling process lowers this antioxidant capability [60]. Therefore, it is necessary to use exogenous antioxidant supplements such as GSH and taurine [60]. The results obtained in this study agreed with the literature on GSH and taurine supplementation, which reported improved sperm motility, viability, and acrosomal reaction in rabbits [22, 41, 61], stallions [62], and goat bucks [63]. Besides, the current findings agreed with the findings of the previous studies, which stated increases of postthaw motility, viability, and acrosomal integrity of ram and mithun (*Bos frontalis*) semen diluted in tris-based extender supplemented with 25 and 50 mM taurine, respectively [40, 64]. Additionally, supplementing 2 mM of reduced glutathione in the cryopreservation media of boar semen improved sperm membrane integrity and postthaw sperm motility [38]. Moreover, using a 4 mM GSH-supplemented extender protected sperm motility, motion kinetics, acrosome integrity, and viability for 24 h 5°C [41]. Our findings disagreed with other authors [42, 65], who reported that GSH and a wide range of taurine supplementation (1.5–50 mM) did not affect rabbit sperm viability, morphology, and acrosomal reaction during cooling. Nevertheless, the reported inferior semen quality in the case of nontreated sperms and small doses of GSH and taurine might reflect the damage to the sperm because of excessive ROS production that exceeds the normal antioxidant potency of sperms [41]. The variations in results between different studies might be due to variations in the reduced levels of normal sperm antioxidant power [66], pH, and osmolality [67]. Besides the supplementation dose, species differences exist in withstanding cryopreservation and cooling-associated stress and thawing-freezing problems [68].

The improving effects of both GSH and taurine may also be due to the prevention of lipid peroxidation, as confirmed by lowering the MDA concentrations in GSH and taurine-treated semen compared to nontreated semen. This effect ascertains the protective role of both taurine and GSH against lipid peroxidation and sperm membrane damage. The high content of polyunsaturated fatty acids (PUFA) in the sperm plasma membrane increases its chance to get deteriorated and damaged, and the consequent release of PUFA, which binds to oxygen, increases ROS production [69]. This finding is possibly explained by increasing the intracellular biosynthesis of GSH, which protects the proteins, DNA, and membrane lipids from ROS-induced damage by the direct radical-scavenging ability of the GSH [70]. The protective effect of GSH and taurine supplementation was also established by lowering the extracellular level of GPT, which is a good indicator of sperm quality, measuring sperm membrane stability [71]. Increasing the percentage of abnormal and dead sperm is usually associated with high levels of GPT in extracellular fluid because the damaged sperm membrane releases these enzymes [72]. Moreover, GSH and taurine were correlated with maintaining fructose levels (all times) relative to the number of live spermatozoa in the extender. Increasing fructose levels at increasing cooling time in nonsupplemented extenders might be linked with a lowering percentage of live spermatozoa compared to the supplementing extenders.

Another interesting finding is that increasing SOD, GPx, and CAT concentrations are linked with increasing the supplementation dose and the cooling time. This finding might explain the improving effect of the exogenous supplementation of GSH and taurine on sperm quality. Cooling and cryopreservation processes impair the sperm's functions and lower its fertilizing capacity by either overproduction of ROS or reducing the antioxidant defense system [60]. Interestingly, the increases in SOD, CAT, and GPx concentrations might be coupled with increasing the antioxidant production by sperm and its release into seminal plasma [73]. This finding confirms that the exogenous GSH and taurine supplementation in semen extender protect the sperm against the excessive production of ROS during chilling by sustaining equilibrium between oxidation and levels of antioxidants [58]. This effect might be correlated with increasing the ability of sperm to synthesize SOD, CAT, and GPx during oxidative stress [39]. This result agrees with other studies that stated an improvement in GSH and SOD activities following GSH supplementation to semen extender as it is a cofactor of the enzymatic antioxidant systems during cooling [41]. The increased levels of antioxidants in the extender might be related to the enhanced transcription levels of its related genes, as confirmed by upregulating the mRNA copies of *CAT* and *SOD* genes. The upregulations of *CAT* and *SOD* following GSH and taurine supplementation to semen extender might be linked to the APS kinase (APK) and glutathione S-transferases (GST) family genes modulation by GSH [74] and to the concentration of taurine in the mitochondria of many cells [75].

Therefore, evaluating the transcriptomic levels of these pathways following taurine and GSH supplementation is recommended. Despite the reported improving effects of GSH and taurine on semen quality, this study has some limitations, including predicting the real male fertility. Also, this study did not highlight the problem of spermatozoa that occurs after entering the female tract. Therefore, other tests such as sperm penetration, sperm viability, and tests examining the sperm's attachment and fertilizing capacity (pregnancy test) are recommended in the future. Moreover, extending this study to cover as long as possible, up to 5-6 days, is recommended.

## 5. Conclusions

Exogenous supplementation of the extender with GSH and taurine improved the quality of rabbit semen cooled at 5°C by increasing the percentage of live spermatozoa, motile sperms, and acrosomal reaction, as confirmed by maintaining fructose level and decreasing GPT in extracellular fluid. Moreover, both GSH and taurine reduced cooling-associated oxidative stress by lowering MDA concentration and increasing SOD, CAT, and GPx levels, along with upregulating the transcription levels of SOD and CAT genes. These improving effects increased with the supplementation dose and the cooling time to 72 h. Therefore, based on these results, the 2 mM GSH and 10 mM taurine can be effectively supplemented with a rabbit's semen extender to enhance the in-vitro sperm quality and antioxidant status of New Zealand rabbits' sperm during chilled storage for up to 72 hours.

## Figures and Tables

**Table 1:** Primer sequences and annealing temperature.

Gene	Primer	Annealing	Slope	Efficiency	Reference
SOD	F: CCCGGTCTTTGTACTCTCGT	60°C + preheating (at 62°C for 1 min)	−3.34	99.25	[46] NM_001082627
	R: AAGGATGAAGAGAGGCACGT				

CAT	F: GCCCTGGTCAGTCTTGTAGT	62°C	−3.42	96.06	[46] XM_002709045
	R: CCATCGGCATATGAACGGAT				

GAPDH	F: ATCTCGCTCCTGGAAGATGG	60°C + preheating (at 62°C for 1 min)	−3.39	97.24	[46] NM_001082253
	R: CAAAGTGGATGTTGTCGCCA				

SOD = superoxide dismutase; CAT = catalase; GAPDH = glyceraldehyde-3-phosphate dehydrogenase (reference gene).

**Table 2:** Effect of different concentrations of glutathione and taurine supplementation (0.5, 1, and 2 mM GSH and 1, 5, and 10 mM taurine) on rabbit sperm motility, vitality, and acrosome reaction rate during 24, 48, and 72 h storage at 5°C.

	Postchilling time (h)	Sperm motility (%)	Sperm vitality (%)	Acrosome reaction rate (%)
*C*	24	87.5 ± 0.50^Aa^	93.00 ± 2.00^Aa^	85.00 ± 0.50^Aa^
	48	74 ± 1.00^Bb^	84.00 ± 4.00^Bb^	67.00 ± 2.00^Ba^
	72	50 ± 5.00^Bc^	77.50 ± 2.50^Cc^	47.50 ± 2.50^Bb^

G0.5	24	88 ± 1.00^Aa^	93.50 ± 1.50^Aa^	88.00 ± 1.00^Aa^
	48	77 ± 2.00^Aa^	86.50 ± 1.50^Aa^	77.50 ± 2.50^ABa^
	72	73 ± 2.00^Ab^	82.5 ± 2.50^Aa^	62.50 ± 2.50^Bb^

G1	24	93 ± 2.00^Aa^	94.00 ± 1.00^Aa^	89.00 ± 2.00^Aa^
	48	82.5 ± 2.50^Aa^	91.50 ± 1.50^Aa^	78.50 ± 6.50^ABa^
	72	66 ± 6.00^Aa^	90.5 ± 2.50^Aa^	67.00 ± 2.00^Aa^

G2	24	93 ± 1.00^Aa^	94.5 ± 0.50^Aa^	93.50 ± 0.50^Aa^
	48	87 ± 2.00^Ab^	92.00 ± 1.00^Bb^	87.00 ± 1.00^ABa^
	72	83 ± 3.00^Ab^	91.00 ± 2.00^Bb^	82.50 ± 2.50^Bb^

T1	24	92.5 ± 2.50^Aa^	93.00 ± 2.00^Aa^	98.50 ± 0.50^Aa^
	48	87.5 ± 0.50^Aa^	91.50 ± 1.50^Aa^	81.00 ± 6.00^Aa^
	72	74.5 ± 1.50^Ab^	89.50 ± 3.50^Ab^	61.00 ± 6.00^Bb^

T5	24	93.5 ± 0.50^Aa^	92.5 ± 2.50^Aa^	89.50 ± 0.50^Aa^
	48	77.5 ± 2.50^Ab^	92.50 ± 0.50^Aa^	83.00 ± 1.00^Aa^
	72	73.5 ± 1.50^Ab^	91.5 ± 1.50^Aa^	72.50 ± 2.50^Aa^

T10	24	91.5 ± 0.50^Aa^	95.00 ± 0.00^Aa^	92.00 ± 1.00^Aa^
	48	84 ± 1.00^Aa^	94.00 ± 1.00^Aa^	88.00 ± 2.00^Aa^
	72	79.5 ± 0.50^Aa^	91.50 ± 3.50^Aa^	82.00 ± 2.50^Aa^

Uppercase and lowercase letters indicate statistical significance at *P* < 0.05 between chilling times and extender supplements (GSH and taurine). C represents the control samples (no treatment), and G1, G2, and G0.5 represent 1, 2, and 0.5 mM of GSH, respectively. T1, T5, and T10 denote 1, 5, and 10 mM of taurine, respectively.

## Data Availability

All data are included in this article.

## Supplementary Materials

Supplementary Materials This figure represents the acrosomal reaction and the sperm's livability. For live and dead sperm, it has been a while since this slide was made, and for that reason, there are no clear differences between the live and dead sperm. In this study, we prepared and examined the slides directly, without taking photos.
