# Supplementary material for: Extender Supplementation with Glutathione (GSH) and Taurine Improves In Vitro Sperm Quality and Antioxidant Status of New Zealand Rabbits during Chilled Storage for up to 72 hours
Source: Vet Med Int. 2023 Sep 12;2023:8339591. doi: 10.1155/2023/8339591 (PMC10509003; doi:10.1155/2023/8339591)
Supplement: Supplementary Materials — This figure represents the acrosomal reaction and the sperm's livability. For live and dead sperm, it has been a while since this slide was made, and for that reason, there are no clear differences between the live and dead sperm. In this study, we prepared and examined the slides directly, without taking photos. [file 8339591.f1.docx]

**Supplementary material**


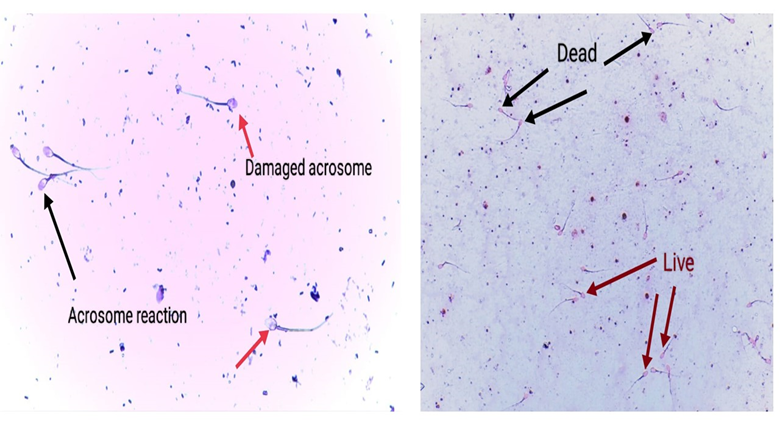


This figure represents the acrosomal reaction and the sperm’s livability. For live and dead sperm, it has been a while since this slide was made and for that reason there is no clear differences between the live and dead sperm. In this study, we prepared and examined the slides directly, without taking photos.
